# Decisional Regret and Long-term Quality of Life After Artificial Urinary Sphincter Implantation Following Radical Prostatectomy

**DOI:** 10.1016/j.euros.2025.12.006

**Published:** 2025-12-26

**Authors:** Ingunn Roth, Christian Beisland, Karin M. Hjelle, Christian Arvei Moen, Elisabeth Grov Beisland, Patrick Juliebø-Jones

**Affiliations:** a Department of Clinical Medicine, University of Bergen, Bergen, Norway; b Department of Urology, Haukeland University Hospital, Bergen, Norway; c Faculty of Health and Social Sciences Western Norway University of Applied Sciences, Bergen, Norway; d The Clinical Research in Aging and NEphro-urology (CRANE) group, Bergen, Norway

**Keywords:** Prostate cancer, Incontinence, Surgery, Quality of life, Regret

## Abstract

**Background and objective:**

Insertion of an artificial urinary sphincter (AUS) is the reference treatment for stress urinary incontinence (SUI) after radical prostatectomy (RP). Although long-term outcomes have been characterised, data on decisional regret remain limited. Our aim was to evaluate decisional regret, quality of life, and the symptom burden among men with an AUS in this setting.

**Methods:**

After ethics approval for the study, all men who had undergone RP and subsequent AUS implantation at a tertiary centre between 2012 and 2023 were identified and contacted by post. The men were invited to complete a series of validated questionnaires: Expanded Prostate Cancer Index Composite (EPIC-26), Decisional Regret Scale (DRS), and Hospital Anxiety and Depression Scale (HADS). The overall response rate was 87.5% (*n* = 91), with median follow-up of 82 mo (interquartile range 49–100).

**Key findings and limitations:**

Pad use significantly improved postoperatively but worsened over time; however, it remained better than at baseline. Higher pad counts correlated with higher depression scores. Overall, 35% of men reported no decisional regret, 34% mild regret, 30% moderate regret, and 1.3% severe regret. Reoperation was the only independent predictor of regret (+18 points on DRS; *p* = 0.001). Better continence scores correlated with lower anxiety and depression, while scores for bowel and hormonal domains also influenced psychological wellbeing.

**Conclusions and clinical implications:**

AUS implantation provides a durable improvement in continence and psychological health for men with post-RP SUI. However, outcomes may deteriorate over time, and the need for reoperation is a significant driver of decisional regret.

**Patient summary:**

We asked men who received an artificial urinary sphincter to treat urinary leakage after prostate surgery to complete questionnaires on their quality of life. Most men reported lasting improvements. Repeat sphincter surgery was linked to more regret, which shows the importance of long-term follow-up and counselling.

## Take Home Message

Implantation of an artificial urinary sphincter (AUS) improves continence and psychological wellbeing in men with postprostatectomy incontinence, but outcomes may decline over time, and repeat AUS surgery strongly predicts decisional regret.

## 1 Introduction

Stress urinary incontinence (SUI) remains a challenging and often distressing complication for men after radical prostatectomy (RP), with incidence rates ranging widely from 4% to 57% [1]. Even mild urine leakage can have a profound effect on health-related quality of life (HRQoL), and can potentially result in social withdrawal, anxiety, and depression. Intimacy and personal relationships can also be affected [2]. Conservative strategies, such as pelvic-floor muscle training, are commonly recommended as first-line therapy. However, the clinical benefit in this particular setting is often limited [3]. For patients with persistent and bothersome SUI, surgical treatment is usually recommended [4], [5]. Among the surgical options available, insertion of an artificial urinary sphincter (AUS) is the reference treatment for moderate to severe SUI. Despite the potential advantages associated with AUS implantation, the decision to proceed with this approach must be carefully weighed against potential drawbacks, such as the risks of surgical complications, mechanical failure, or a need for device removal. Both the European Association of Urology and American Urological Association guidelines highlight the importance of comprehensive preoperative counselling to make patients aware of these potential risks and manage expectations accordingly [6], [7].

An understanding of patient-centred outcomes, including decisional regret, is essential when evaluating the long-term success of any SUI intervention such as AUS surgery. Greater knowledge of potential predictors of regret could support preoperative counselling and improve the shared decision-making (SDM) process, and help in ensuring that surgery aligns with the unique treatment goals of each individual patient. The residual symptom burden, whether arising from persistent incontinence or device-related complications, may substantially affect both patient satisfaction and the perceived benefit of the implant surgery [8].

The aim of this study was to evaluate the relationship between symptom burden, HRQoL, and decisional regret at long-term follow-up for men who underwent AUS implantation for SUI after RP to inform strategies for counselling, patient selection, and postoperative support.

## 2 Patients and methods

**Fig. 1:**
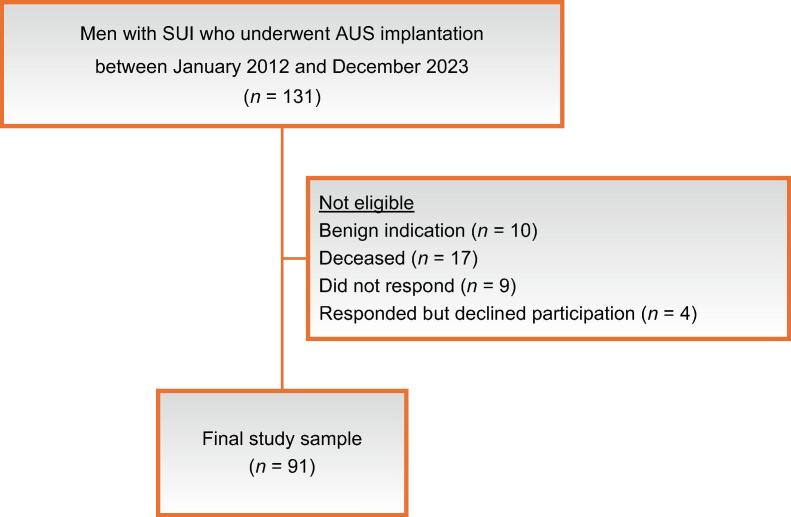
Flow chart of the study inclusion process. AUS = artificial urinary sphincter; SUI = stress urinary incontinence.

All men who had undergone implantation of an AMS 800 AUS device between January 2012 and April 2023 at Haukeland University Hospital, a tertiary centre in Western Norway, were identified. Inclusion criteria were: (1) still alive at the time of the study and (2) SUI as a sequela of RP. On the basis of these criteria, 104 men were eligible (Fig. 1). All participants were contacted by post, invited to participate in the study, and sent a series of questionnaires. This occurred in late 2024, after a minimum follow-up period of 18 mo for every participant. In the absence of a postal response, a single telephone call was placed to verify receipt of the survey. It was explicitly stated in the accompanying information that the project concerned the implant surgery, and that the questions regarding decisional regret referred specifically to this procedure rather than to the patient’s original choice of prostate cancer treatment.

The overall response rate was 87.5% (*n* = 91). Median study follow-up was 82 mo (interquartile range [IQR] 49–100). The validated questionnaires used were the Expanded Prostate Cancer Index Composite Short Form (EPIC-26) [9], the Decisional Regret Scale (DRS) [10], and the Hospital Anxiety and Depression Scale (HADS) [11]. Permission for use of each questionnaire in the research setting was obtained from the relevant bodies, as required. For each instrument, raw scores were converted according to the official scoring guidelines provided by the respective developers. For EPIC-26, the five domain scores were transformed to a scale from 0 to 100 scale, where 0 represents the worst possible symptoms and 100 indicates no symptoms. The DRS was also scored on a scale from 0 to 100, with higher scores reflecting greater regret. Following a classification used by other research groups, DRS scores were categorised as follows: 0 = no regret, 1–25 = mild regret, 26–65 = moderate regret, and 66–100 = severe regret.

The HADS is recommended for assessing psychological distress in the population aged 65–80 yr [12]. The instrument consists of 14 items for two subscales (one for anxiety and one for depression), each of which has a maximum score of 21 [13]. A score of ≥8 indicates a possible case of anxiety or depression [14]. Baseline data were also collected, including demographics and clinical characteristics such as prior radiotherapy, comorbidities (eg, diabetes), American Society of Anesthesiologists physical status, and age at primary prostate surgery. Data for the diagnostic workup, operative technique, and early postoperative outcomes, including complication rates and a need for reoperation, have been reported in an earlier study [15].

### 2.1 Statistical analysis

Spearman’s rank correlation was applied to assess associations between EPIC-26 domain scores and psychological measures (HADS, DRS), as the data related to ordinal variables that were not normally distributed. Simple and multivariable linear regression analyses were performed to assess the predictive impact of factors such as pad use, age, body mass index (BMI), and repeat surgery. The EPIC-26 urinary incontinence item for pad use, which categorises responses into four ordinal groups (no pads, 1 pad/d, 2 pads/d, and ≥3 pads/d) was applied at three time points: before AUS surgery (T1), after AUS surgery (T2), and at study follow-up (T3). Descriptive statistics are used to summarise pad use at each time point. Within-patient changes in pad use were analysed using Wilcoxon signed-rank tests for the three planned pairwise comparisons: T1 versus T2, T2 versus T3, and T1 versus T3. A subgroup analysis was performed to determine whether participants who experienced severe worsening, defined as an increase in pad use of two or more categories from time T2 to T3, had higher odds of decisional regret on logistic regression. Analysis for the nonresponder group was used to compare baseline parameters. For this analysis, the Mann-Whitney test was used for continuous variables given the lack of normal distribution, while Fischer’s exact test was applied for categorical variables given the low cell count. All statistical analyses were performed using IBM SPSS v29.0, with statistical significance set at *p* < 0.05. The study was approved by the regional ethics committee of Western Norway (REK2024/726791).

## 3 Results

### 3.1 Patient characteristics

The final sample included 91 patients with a median age of 69 yr (IQR 65–72) at the time of their AUS surgery. Previous radiotherapy had been performed in 34% (*n* = 31) of patients, and 49.5% (*n* = 45) had a history of smoking (Table 1). Severe urinary incontinence, classified as >400 g/24 h, was present at the time of AUS implantation in 63.7% of patients, with a median pad count of 5/d (IQR 4–6). During the follow-up period, 25.3% (*n* = 23) of the patients underwent repeat surgery (Supplementary Table 1).

**Table 1:** Baseline characteristics of the study cohort (*n* = 91)

Parameter	Result
Index surgery, *n* (%)	
Robot-assisted laparoscopic prostatectomy	76 (83.5)
Open radical retropubic prostatectomy	13 (14.3)
Salvage prostatectomy	2 (2.2)
Prior radiotherapy, *n* (%)	31 (34)
Comorbidities, *n* (%)	
Hypertension	43 (47.3)
Diabetes mellitus	9 (9.9)
History of smoking, *n* (%)	45 (49.5)
Median age at prostate surgery, yr (IQR)	64 (61–67)
Median age at AUS insertion, yr (IQR)	69 (65–72)
Median ASA score (IQR)	2 (2–2)
Median body mass index, kg/m^2^ (IQR)	27.5 (25.7–29.9)
Previous incontinence surgery, *n* (%)	13 (14.3)
Degree of leakage, *n* (%)	
Mild (<100 g/24 h)	3 (3.3)
Moderate (100–400 g/24 h)	22 (24.2)
Severe (>400 g/24 h)	58 (63.7)
Data missing	8 (8.8)
Median number of pads/24 h (IQR)	5 (4–6)
Median follow-up, mo (IQR)	82 (49–100)
Need for reoperation, *n* (%)	23 (25.3)

IQR = interquartile range; ASA = American Society of Anesthesiologists; AUS = artificial urinary sphincter.

### 3.2 Predictors of decisional regret

Overall, 35% of the respondents had zero decisional regret, while 33.8% had mild and 30% had moderate regret. Severe regret was rare, reported by only 1.3% of the cohort (Table 2). On multivariable linear regression, repeat surgery emerged as the only significant predictor of decisional regret. Patients who underwent repeat surgery had, on average, an 18.0-point higher regret score (B = 18.0, 95% confidence interval [CI] 7.2–28.8, *p* = 0.001) in comparison to those without repeat surgery. Age, BMI, and the number of pads used per day were not significant predictors.

**Table 2:** Summary of questionnaire scores

Instrument	Result
Median EPIC-26 domain summary score (IQR)	
Urinary: incontinence	52 (23–74)
Urinary: irritative/obstructive	81 (69–94)
Bowel	94 (75–100)
Sexual	8 (0–17)
Hormonal	85 (70–100)
Decisional regret (%)	
No regret (score 0)	35
Mild regret (score 1–25)	33.7
Moderate regret (score 26–65)	30
Severe regret (score 66–100)	1.3
Median HADS score (IQR)	
HADS-Anxiety	2.5 (0.75–7)
HADS-Depression	3 (1–6.25)

EPIC-26 = Expanded Prostate Cancer Index Composite; HADS = Hospital Anxiety and Depression Scale; IQR = interquartile range.

On the basis of the number of events, additional covariates were not included in the multivariable model to preserve its stability. Potential covariates, such as prior sling status, previous radiotherapy, and device size, were examined in univariate analyses, but none reached statistical significance.

### 3.3 Urinary incontinence, HADS scores, and pad use

According to results for the urinary incontinence (UI) domain of the EPIC-26 questionnaire, there was a moderate negative correlation between UI scores and decisional satisfaction (ρ = –0.379, *p* < 0.001), which indicates that better UI scores were associated with higher decisional satisfaction. Better UI scores were also associated with lower anxiety (ρ = −0.259, *p* = 0.019) and lower depression scores (ρ = −0.359, *p* < 0.001). Pad use was positively correlated with depression (ρ = +0.289, *p* = 0.007). Simple linear regression analysis revealed that each additional pad used per day was associated with a 1.24-point increase in depression score (*p* = 0.004) and a 0.58-point increase in anxiety score (*p* = 0.175), although the latter did not reach statistical significance.

### 3.4 Other EPIC-26 domains and HADS scores

Analysis of other EPIC-26 domains in relation to HADS scores revealed a negative association between bowel domain scores and both anxiety (ρ = −0.467, *p* < 0.001) and depression (ρ = −0.541, *p* < 0.001). Hormonal health scores exhibited a strong negative correlation with anxiety (ρ = −0.526, *p* < 0.001) and depression (ρ = −0.606, *p* < 0.001). By contrast, there was no significant correlation between sexual function scores and either of the HADS domains.

### 3.5 Change in pad count over time

Pad use declined markedly from before AUS surgery (T1) to the postoperative assessment (T2): 93.8% of patients were using ≥3 pads/d at baseline, while 60.8% were pad-free and 31.6% using 1 pad/d postoperatively (Table 3). Wilcoxon signed-rank tests revealed a significant reduction in pad use from T1 to T2 (*Z* = −7.405, *p* < 0.001, *r* = 0.87).

**Table 3:** Pad count across study time points

Pad count	Time point		
	Before surgery (T1)	3 mo after surgery (T2)	Follow-up (T3)
0 pads (%)	0	60.8%	16.5%
1 pad (%)	1.2%	31.6%	42.9%
2 pads (%)	4.9%	2.5%	23.1%
≥3 pads (%)	93.8%	5.1%	17.6%
Median pads used, *n* (IQR)	3 (3–3)	0 (0–1)	1 (1–2)

Between T2 and follow-up (T3), pad use increased, with the proportion of pad-free patients falling to 16.5% and a greater number of patients using ≥2 pads/d. This deterioration was statistically significant (*Z* = 7.307, *p* < 0.001, *r* = 0.82).

Comparison of T1 versus T3 showed that pad use remained significantly improved relative to baseline (*Z* = 7.244, *p* < 0.001, *r* = 0.80), although the magnitude of the benefit was less than that observed in the immediate postoperative period (Table 3).

Worsening of pad use by two or more categories from T2 to T3 was not significantly associated with decisional regret (odds ratio 1.27, 95% CI 0.34–4.81; *p* = 0.73).

### 3.6 Analysis of the nonresponder group

Comparison of baseline characteristics for the responder and nonresponder groups revealed that mean baseline pad use was 5.3 (standard deviation [SD] 2.4) in both groups (*p* = 0.98). Mean age was 69 yr (SD 10) for nonresponders and 69 yr (SD 5.5) for responders (*p* = 0.75). There was no significant difference in the frequency of prior radiotherapy (30.8% vs 34.1%; *p* = 1.00) or smoking (69% vs 49.5%; *p* = 0.24) between the groups. Mean BMI (SD) was higher in the nonresponder group (30 kg/m^2^, SD 4.2) than in the responder group (28 kg/m^2^, SD 3.4; *p* = 0.03).

Although not a baseline characteristic, reoperation was more frequent among non-responders (46.2%) than responders (25.3%; p = 0.12).

## 4 Discussion

Long-term follow-up after AUS implantation for SUI after RP revealed that the majority of patients reported no or only mild decisional regret. These findings align with previous reports indicating that AUS surgery can provide substantial HRQoL benefits, even in the presence of persistent incontinence [1]. The strongest predictor of decisional regret in our cohort was a need for repeat surgery. Previous studies have also found that patient dissatisfaction is a strong predictor of decisional regret [1]. This underscores the importance of counselling patients about the likelihood of device revision as part of the preoperative decision-making process. There has been increasing emphasis on SDM in recent years, and Hampson et al [16] demonstrated that higher SDM scores were an independent predictor of lower decisional regret. A qualitative study by the same group highlighted that complex UI treatment choices require an individualised approach, as the traditional endpoint of achieving dryness may not reflect what every patient considers to be the most important attribute [17].

Regarding other potential predictors of regret, our study did not identify any that were statistically significant. Participants with greater worsening of pad burden between initial and long-term follow-up had higher odds of regret, but this association did not reach statistical significance. A larger sample size might have provided sufficient power to detect such an effect. Similarly, this pattern might be anticipated among individuals who experience a comparable decline after previous sling surgery before subsequent AUS implantation. Beyond the factors evaluated in this study, preoperative psychological characteristics such as personality traits and baseline mood deserve consideration. For example, in a study of women who underwent reconstruction surgery after mastectomy for breast cancer, higher levels of preoperative depression, anxiety, and stress predicted higher levels of decisional regret at follow up [18]. Such factors could be relevant in the context of UI surgery after RP.

In our study, an improvement in continence translated to measurable psychological benefits, as better UI scores were associated with an improvement in mental wellbeing. A higher pad count was associated with higher levels of depressive symptoms, consistent with previous studies on persistent UI following surgery [16]. These findings support the role of continence not only as a functional outcome but also as a determinant of mental wellbeing. Addressing patients’ psychological health may enhance participation in treatment decisions and satisfaction with care.

Although the continence burden remained improved in comparison to baseline, the degree of improvement at T3 had diminished since the initial postoperative follow-up. This gradual decline may be attributable to device wear, urethral atrophy, or age-related changes. Our findings underscore the complex interplay between functional outcomes, psychological health, and treatment satisfaction. Although most patients experience a durable benefit from AUS surgery, residual or recurrent symptoms, together with the risk of repeat surgery, may adversely affect long-term satisfaction.

### 4.1 Strengths and limitations

Strengths of this study include the high response rate, long median follow-up, and use of validated patient-reported outcome measures, with participation achieved without monetary incentives. Limitations include the absence of baseline psychological data, which precludes assessment of longitudinal changes in mental health, and the single-centre design, which may limit generalisability. While baseline characteristics were largely comparable between the responder and nonresponder groups, BMI was higher in the latter, and reoperation rates also differed between the groups. Although reoperation is not a baseline characteristic, these differences may indicate a degree of selection bias and should be considered when interpreting the results. The relatively small sample size may also have limited statistical power, and some comparisons may therefore not have reached significance. An additional limitation is that data for clinical outcomes, such as prostate cancer recurrence and the use of androgen deprivation therapy, were not collected. Future studies should assess the impact of AUS implant surgery on HRQoL in a prospective setting and integrate the use of patient-reported outcome measures in routine clinical practice. Although the global volume of surgeries for SUI after RP is substantial, the corresponding literature on patient wellbeing remains very limited.

## 5 Conclusions

AUS implantation can provide lasting improvements in continence for men with SUI after RP. However, these benefits may gradually decline over time. While most patients do not regret undergoing incontinence surgery, the need for repeat surgery is a significant contributory factor for those who later experience decisional regret.

  ***Author contributions***: Patrick Juliebø-Jones had full access to all the data in the study and takes responsibility for the integrity of the data and the accuracy of the data analysis.

  *Study concept and design*: Roth, Juliebø-Jones, Beisland.

*Acquisition of data*: Roth, Moen, Hjelle, Juliebø-Jones.

*Analysis and interpretation of data*: Roth, Beisland, Hjelle, Moen, Grov Beisland, Juliebø-Jones.

*Drafting of the manuscript*: Roth, Juliebø-Jones.

*Critical revision of the manuscript for important intellectual content*: Roth, Beisland, Hjelle, Moen, Grov Beisland, Juliebø-Jones.

*Statistical analysis*: Roth, Juliebø-Jones.

*Obtaining funding*: None.

*Administrative, technical, or material support*: None.

*Supervision*: Juliebø-Jones, Beisland.

*Other*: None.

  ***Financial disclosures:*** Patrick Juliebø-Jones certifies that all conflicts of interest, including specific financial interests and relationships and affiliations relevant to the subject matter or materials discussed in the manuscript (eg, employment/affiliation, grants or funding, consultancies, honoraria, stock ownership or options, expert testimony, royalties, or patents filed, received, or pending), are the following: None.

  ***Funding/Support and role of the sponsor*:** None.

  ***Ethics statement:*** This study was approved by the regional ethics committee of Western Norway (REK2024/726791).

  ***Data sharing statement*:** The data sets generated during and/or analysed during the current study are available from the corresponding author on reasonable request.
