# Supplementary material for: Decisional Regret and Long-term Quality of Life After Artificial Urinary Sphincter Implantation Following Radical Prostatectomy
Source: Eur Urol Open Sci. 2025 Dec 26;83:185–90. doi: 10.1016/j.euros.2025.12.006 (PMC12796753; doi:10.1016/j.euros.2025.12.006)
Supplement: Supplementary Data 1 [file mmc1.docx]

| **Reoperation type** | **n (%)** |
| --- | --- |
| Hematoma: reoperation the same day | 1 (4.3) |
| Infection in perineal incision; revision | 1 (4.3) |
| Balloon misplaced; repositioned | 1 (4.3) |
| Pain due to pump; repositioned | 1 (4.3) |
| Infection; removal of AUS | 2 (8.7) |
| Erosion of cuff; removal of AUS | 8 (35) |
| Mechanical failure; removal AUS and implant AUS | 4 (17.4) |
| Increasing incontinence; tandem cuff | 4 (17.4) |
| Increasing incontinence; replacement of cuff | 1 (4.3) |

**Supplementary Table 1.** Summary of reoperation data

AUS = Artificial urinary sphincter
